# Face and Voice as Social Stimuli Enhance Differential Physiological Responding in a Concealed Information Test

**DOI:** 10.3389/fpsyg.2012.00510

**Published:** 2012-11-19

**Authors:** Wolfgang Ambach, Birthe Assmann, Bennet Krieg, Dieter Vaitl

**Affiliations:** ^1^Institute for Frontier Areas of Psychology and Mental HealthFreiburg, Germany; ^2^Institute of Biology, Freie Universität BerlinBerlin, Germany; ^3^Bender Institute of Neuroimaging, University of GiessenGiessen, Germany

**Keywords:** Concealed Information Test, deception, mock-crime, social stimuli

## Abstract

Attentional, intentional, and motivational factors are known to influence the physiological responses in a Concealed Information Test (CIT). Although concealing information is essentially a social action closely related to motivation, CIT studies typically rely on testing participants in an environment lacking of social stimuli: subjects interact with a computer while sitting alone in an experimental room. To address this gap, we examined the influence of social stimuli on the physiological responses in a CIT. Seventy-one participants underwent a mock-crime experiment with a modified CIT. In a between-subjects design, subjects were either questioned acoustically by a pre-recorded male voice presented together with a virtual male experimenter’s uniform face or by a text field on the screen, which displayed the question devoid of face and voice. Electrodermal activity (EDA), respiration line length (RLL), phasic heart rate (pHR), and finger pulse waveform length (FPWL) were registered. The Psychopathic Personality Inventory – Revised (*PPI-R*) was administered in addition. The differential responses of RLL, pHR, and FPWL to *probe* vs. *irrelevant* items were greater in the condition with social stimuli than in the text condition; interestingly, the differential responses of EDA did not differ between conditions. No modulatory influence of the *PPI-R* sum or subscale scores was found. The results emphasize the relevance of social aspects in the process of concealing information and in its detection. Attentional demands as well as the participants’ motivation to avoid detection might be the important links between social stimuli and physiological responses in the CIT.

## Introduction

### The concealed information test

Concealing information from an interrogator is a specific social behavior commonly performed by a culprit in order to hide his or her involvement in a criminal act. A scientific psychophysiological method to detect intentionally hidden information is the Concealed Information Test (CIT), which combines a systematic interrogation with a simultaneous measurement of several physiological data channels. The core assumption of the CIT is that a guilty subject’s physiological responses are different for crime-related information compared to crime-irrelevant information (Lykken, 1959). The CIT consists of several multiple-choice questions each referring to another detail of the crime under investigation. Typically, there are four to five answer alternatives to each question but only one alternative, the “probe,” refers to the critical detail. For example, if an envelope was stolen out of an office, a typical CIT question could be “An office requisite has been stolen. Is this the stolen object?”; this question is combined with a sequence of five pictures representing the respective answer alternatives, e.g., a picture of (a) a pencil sharpener, (b) an envelope, (c) a highlighter, (d) a stapler, and (e) a Scotch® Tape. In this example, the picture of the envelope (b) is the “probe” item; the other items are referred to as “irrelevant.” It is assumed that only subjects possessing crime-related knowledge (“guilty” subjects) will recognize the correct item and show a different physiological response to it. Subjects without such knowledge (“innocents”) cannot discriminate between the *probe* and *irrelevant* alternatives and therefore will not show a systematic response pattern. Numerous laboratory studies have shown that the CIT is a highly valid test for differentiating between guilty and innocent subjects (for a review see Ben-Shakhar and Elaad, 2003).

Concealed Information Test theory is heavily based on cognitive approaches, particularly the orienting response (Sokolov, 1963; Lykken, 1974). While motivational and emotional influences are thought to play a minor, only mediating role in laboratory CIT experiments, their importance might well be enhanced in field examinations (Verschuere and Ben-Shakhar, 2011). So far, the qualitative and quantitative differences in attentional, intentional, motivational, emotional, and social factors influencing the CIT in laboratory and field situations are only barely understood. CIT mechanisms that go beyond the orienting reflex merit more attention; the relation between social situation and physiological responding in the CIT still has to be elaborated.

### Social aspects and the CIT

Within the last decades, the social aspects of concealing information have played only a minor role in CIT research. As a predominant trend occurring in parallel, the participants in laboratory CIT experiments were mostly seated alone in an experimental chamber and the former “interrogator” was replaced by an interrogative computer interface to interact with. The availability of computerized experimental methods supported this change in CIT research. By minimizing uncontrolled social influences, particularly by the experimenter (Iacono, 2000), it became possible to standardize CIT experiments to a certain degree. Yet, in the course of this trend, the social aspects of withholding information have faded into the background, although information concealment is essentially a social action.

Earlier studies focused on the social influence on physiological responding in the CIT questioning situation (Orne, 1975; Waid and Orne, 1981; see also Iacono, 2000). Yet, neither social interactions, nor social roles, nor the presence of social stimuli were systematically varied in these studies.

The differential responses to *probe* vs. *irrelevant* items in a CIT are known to be influenced by attentional, intentional, and motivational factors: a greater motivation to remain undetected is related to greater differential responding (Gustafson and Orne, 1963; Elaad and Ben-Shakhar, 1989; Furedy and Ben-Shakhar, 1991; Ben-Shakhar and Elaad, 2003). Likewise, a demonstration of the effectiveness of the apparative detection procedure enhances the physiological response differences (Stern et al., 1981; Saxe, 1991), as does a lack of perceived success in deceiving (Gustafson and Orne, 1965). The same holds for a stronger intention to deceive (Furedy and Ben-Shakhar, 1991), a greater response conflict between the predominant truthful and the required deceptive answer (Furedy and Ben-Shakhar, 1991; Bradley et al., 1996), and a greater attentiveness throughout the test (countered by countermeasures; see, e.g., Elaad and Ben-Shakhar, 1991). In addition, an “active” questioning format (e.g., “Did you steal this object?”) has been suggested to be more effective than a more “passive” questioning format (e.g., “Was this object in the deed room?”, “Did you see this object?”; Bradley et al., 1996; Ambach et al., 2011a; but see Gamer, 2010).

It is conceivable that several of these factors that influence differential responding in a CIT depend on the social situation in which the CIT takes place. For example, the physical presence of an interrogator might enhance the motivation to remain undetected or the fear of being detected; on the contrary, facilitating the motivation to confess is also conceivable; a combination of both might enhance response conflict. Participants might also perceive the interrogator as controlling their behavior in the CIT, which would help to focus attention on the test; on the other hand, a present interrogator might divert attention from the test. The presence of a person might also lead to a stronger emotional involvement in the situation and to a more intense conflict between disclosure and withholding information, i.e., between truthful and deceptive responding; a tendency toward withdrawal and alienation, i.e., lower emotional involvement, is thinkable as the opposite. While both directions of influence are principally conceivable, more general studies on the social influences on physiology predominantly suggest an increased involvement and enhanced physiological responding in a more “social” condition:

A general dependence of physiological responses on social aspects, particularly the presence of another person, is assumed due to the findings of earlier sociophysiological studies. Zajonc (1965) derived his *social facilitation theory* from studies investigating the influence of the sheer presence of another person (“audience”) and the “co-action” with another person on a subject’s behavior; increased arousal, “stress,” and induced emotions (e.g., fear) were assumed to be important moderators of behavior and physiological correlates. Martens (1969) found palmar sweating increased when subjects learned a motor task in the presence of an audience as compared to learning the same task alone. Glass et al. (1970) found greater skin conductance levels (SCL) in participants watching an aversive film if they were accompanied by a second spectator. Apprehension about evaluation, i.e., the presence of an evaluative second person, has been shown to increase muscle tension (Chapman, 1973) as well as heart rate (HR; Hrycaiko and Hrycaiko, 1980) as indicators of arousal. Referring to the CIT, the social situation, under which the test is applied, is supposed to comprise aspects of (negative) social evaluation and enhanced negative emotions (e.g., guilt, fear), which increase stress and arousal in an individual.

If the social conditions, under which a participant is investigated in the CIT, are influencing the various physiological responses, another question immediately arises: which components of the social situation are crucial for influencing attention, intention, motivation, emotion, and the accompanying physiological responses in a CIT? Beyond the evidence that the sheer presence of a second person can influence behavior and physiology, the type of social interaction, and specific social elements in a given situation have proven important: negative social evaluation specifically increases salivary cortisol levels (Dickerson et al., 2008). Specific interaction with virtual others has been observed to lead to brain activity different to that induced by the mere presence of virtual others (Schilbach et al., 2006). Considering observable behavior, Haley and Fessler (2005) found that a picture with a pair of eyes increased generosity in an anonymous game. In a study by Sproull et al. (1996), a virtual “talking face,” in contrast to a “text display,” made participants more aroused and led them to present themselves in a more positive light.

In sum, specific situational components of a social interrogation (which the original CIT is) influence emotions, arousal, and motivation of a participant. Visual (i.e., seeing a face or parts of it) and auditory elements (i.e., hearing a voice) make a computer interface more human-like and can, thus, be assumed to induce behavior and physiology more similar to a real-life interpersonal interrogation. While some studies used an auditory presentation of the CIT questions, others used a text display; to our knowledge, a comparison of both has not yet been undertaken. In addition, so far no CIT studies exist employing other social stimuli like a virtual investigator’s face within a virtual interrogation situation.

### Personality aspects in the CIT

Differential psychology in the context of the CIT has been studied since the very origin of the test; yet, various questions still remain open. First, physiological responding strongly differs between individuals; differences in electrodermal lability or HR variability have been shown to be associated with personality traits such as neuroticism, extraversion, and impulsivity (Coles et al., 1971; Crider and Lunn, 1971; O’Gorman, 1990). Lykken (1957) found lower overall electrodermal response amplitudes in sociopathic individuals. Later studies found personality traits such as the “level of socialization” (Waid, 1976; Waid et al., 1979; Waid and Orne, 1980, 1981) to be correlated with differential physiological responding in the CIT and the detection of deception in general.

Over the last decades, psychopathy has been a prominent personality concept in this line of research. An established assessment instrument for psychopathy, even in a standard population sample, is the Psychopathic Personality Inventory – Revised (*PPI-R*; Lilienfeld and Andrews, 1996; Lilienfeld and Widows, 2005; German version: Alpers and Eisenbarth, 2008). Its relation to individual differences in physiological responding to CIT items has repeatedly been studied, particularly from a forensic perspective. Accordingly, most of the research exclusively used male participants and followed the standard computer-based interrogation procedure. As summarized by Verschuere (2011), the so far reported studies investigating CIT accuracy in samples differing with respect to delinquency and psychopathy have yielded inconsistent results. While some studies (e.g., Verschuere et al., 2007) report reduced overall electrodermal responding in prison samples, others (e.g., Verschuere et al., 2005) do not; a solid correlation between psychopathy score and differential responding in the CIT cannot be regarded as confirmed. In sum, personality influences on physiological responding in the CIT need to be elucidated by further research.

In connection with the main focus of the study, we were particularly interested in a psychopathy measure, because psychopathy, repeatedly described as including an “affective, interpersonal facet” (summarized by Verschuere, 2011), can be assumed to influence social interaction and possibly its physiological correlates. Social stimuli might exert different impact in individuals with different psychopathy scores. With respect to the CIT, it is speculatively questioned whether the influence of a present person or other social stimuli might be modulated by specific personality traits such as psychopathy.

### Aim of the present study

1. We examined the influence of face and voice as social stimuli on the physiological responses in a CIT. In this first attempt to directly examine this influence, we did not aim at differentiating between the modality of question presentation (visual vs. auditory) and its social content. Rather, in order to maximize the effect of “text” vs. “face and voice” presentation, we employed a uniform male face in combination with a neutral but serious male voice to simulate the “virtual investigator.” In a between-subjects manipulation, two CIT variants, a “text” and a “social” condition, were compared with respect to differential physiological responding. We expected greater differential responses and higher correct-classification rates in the condition with social stimuli (as compared to the “text” condition) for all physiological measures.
2. We included the *PPI-R* questionnaire in order to investigate whether differential physiological responding in a CIT is mediated by psychopathic traits, i.e., whether participants with higher *PPI-R* sum scores show smaller differential physiological responses. An analysis of correlation coefficients between physiological response differences and the *PPI-R* scores was planned for this purpose. Additionally, we were interested in possible interactions between the influence of social stimuli and the psychopathy score: we expected the influence of social stimuli on differential physiological responding to decrease with heightened *PPI-R* scores.

## Materials and Methods

### Subjects

Seventy-one healthy students (33 males, 38 females; mean age 23.4 ± 3.7 years) voluntarily participated in the study. They were paid 12 Euros, with an additional incentive of 3 Euros. Data from two subjects were discarded from evaluation because of technical problems or insufficient compliance with the instructions. An ethics committee confirmed that the study met all ethical requirements.

### Design and procedure

The experiment was divided into two parts (mock-crime in an “office room” and detection procedure in the “laboratory”), each guided by a different experimenter.

To begin with, the first experimenter explained the procedure to the subjects in the reception room of the department; informed consent was obtained from all participants. A cover story and the use of two rolled-up documents were used to make participants believe that they randomly drew one of two different instructions to perform a “special task” in the first part of the experiment, while, in fact they all received an equivalent mock-crime instruction. The second experimenter, who (in accordance with the information given to the participants) was blind with respect to the mock-crime objects a particular participant had handled in the first part, was introduced as the person responsible for “detecting whether the subjects had stolen something in the office room or not.” Subjects were randomly assigned to either of two groups: half of the subjects (i.e., the *text* group; 34 valid data sets) underwent a CIT using questions presented within a text field on the screen. The other half (i.e., the *social* group; 35 valid data sets) underwent a CIT with questions being asked by a pre-recorded male voice presented via loudspeakers, while a male face was presented as a picture on the screen. Written CIT instructions for the *text* group stated that the experimenter’s aim was to find out the truth by means of “a computer program and physiological measurement,” whereas the corresponding instructions for the *social* group stated that the experimenter’s aim was to find out the truth by means of “a virtual investigator and physiological measurement.” After completing the CIT and a subsequent memory test, subjects filled in the *Psychopathic PPI-R* before they were debriefed and released. Payment included the incentive of 3 Euros, regardless of a participant’s responding in the CIT.

### Mock-crime scenario

Alone and unwatched in an office room of the institute, subjects unrolled the “task instruction” obtained from the first experimenter. They had to remove (“steal”) nine objects from this room after having extensively viewed each of them. The choice of the nine objects, one from each category, was randomized and balanced across subjects. The object categories, each comprising five objects, were: key pendants, kitchen objects, boxes, office materials, cosmetics, wooden toy fruits, drink packages, playing cards, and plastic flowers.

Subjects were advised to collect all nine items in a suitcase, which they should keep closely to themselves throughout the remaining experiment. An amount of 3 Euros was hidden in one of the stolen objects (a box); later, this served as an incentive to “remain undetected.”

### Concealed information test

The “physiological investigation” took place in the laboratory with the second experimenter; recording devices were attached. The CIT consisted of nine blocks referring to the nine item categories (e.g., key pendants, cosmetics). Each block comprised one question with five answer alternatives: the *probe* (“stolen”) item of each category and four corresponding *irrelevant* items, which were all unknown to the subjects.

**Figure 1:**
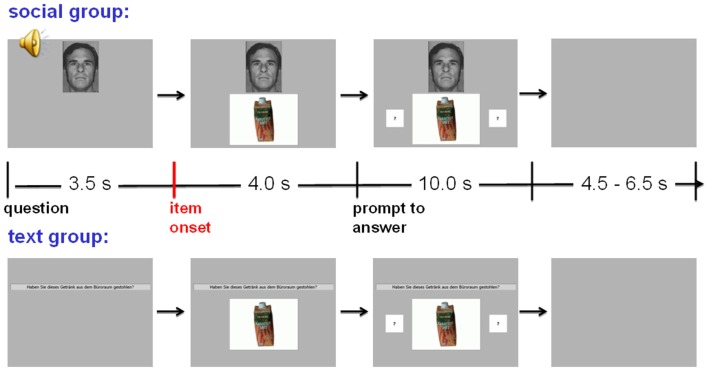
**Question and item presentation in the Concealed Information Test in the *text* group and the *social* group**. Question text or face and voice, respectively, appeared first, CIT items appeared 3.5 s later, and fields with question marks succeeded 4.0 s thereafter. After the key press, a “yes” or “no” text (reflecting the subject’s answer) replaced the question marks. (Translation of the German question text: “Did you steal this wooden fruit from the office room?”)

For the *text* group, the text of each question appeared on the screen five times in sequence, each time followed by a different picture of one of the five answer alternatives, which appeared below the question with a delay of 3.5 s. For the *social* group, the question was presented acoustically with a pre-recorded male voice via speakers; instead of a written question, the picture of “the investigator,” a uniform male face, appeared on the screen. This picture was derived from an Ekman picture in black and white; the man’s facial expression was serious and he was about 40 years old. Figure 1 shows pictures of the screen for both groups in the phase after a specific item was presented, but before the answer was given.

The first item presented for each question served as buffer item; the according trials were discarded from analysis. Preceding each block, two *neutral* items were presented as distractors. The according questions referred to everyday objects that had to be identified (e.g., “Is this a slide projector?”). The two questions had to be answered correctly, one with “yes” and the other with “no” (in a pseudorandomized sequence), to prevent subjects from answering automatically with “no.” Responses to these *neutral* questions were not evaluated. Together with the two *neutral* questions preceding each category, the entire procedure resulted in a total of 63 item presentations. The main run was preceded by a training run consisting of two blocks, each with five *neutral* items. Questions and item pictures were presented for 10 s foveally on a 19″ monitor at a distance of 90 cm, followed by a blank screen for equally distributed 4.5–6.5 s intervals. Picture size was 10.6° by 8.0° of visual angle for the CIT items; the “investigator” presented in the *social* group was 5.6° by 8.0° in size. Four seconds after a question was asked, two indication fields containing question marks appeared on either side of the item picture; this prompted the subjects to answer. Then, answers had to be given as quickly as possible by pressing one of the two response keys and by vocally responding with “yes” or “no.” Key assignment was balanced across subjects. Following the answer, the given “yes” or “no” replaced the question marks and remained visible on the screen as long as the item question was presented.

Subjects were told to hide their knowledge about the objects that had been stolen from the administration room, i.e., to deny all knowledge about *probe* items. Different from the typical CIT wording, an active questioning format was chosen: questions were, e.g., “Did you steal this cosmetic product from the administration room?”

After subjects were disconnected from the leads, they underwent a memory test: all five pictures of each category were presented on the screen simultaneously, one item category after the other; subjects were asked to identify the item they had stolen within each category.

### Physiological measures

The physiological recordings took place in a dimly lit, electrically and acoustically shielded experimental chamber (*Industrial Acoustics GmbH, Niederkrüchten, Germany*). Subjects sat in an upright position so that they could comfortably see the monitor and reach the keyboard. Temperature in the cabin was set to 21°C at the beginning of the first run, with an increase of maximum 2°C throughout the course of the experiment.

Skin conductance, respiratory activity, electrocardiogram (ECG), and finger plethysmogram were registered. Physiological measures were A/D-converted and logged by the *Physiological Data System I 410-BCS* manufactured by *J&J engineering (Poulsbo, WA, USA)*. The A/D-converting resolution was 14 bit, allowing skin conductance to be measured with a resolution of 0.01 μS. All data were sampled with 510 Hz. Triggers indicating question onsets were registered with the same sampling frequency.

For skin conductance recordings, standard Ag/AgCl electrodes (*Hellige*; diameter 0.8 cm), electrode paste of 0.5% saline in a neutral base (*TD 246 Skin Resistance, Mansfield R&D, St. Albans, Vermont, UK*), and a constant voltage of 0.5 V were used. The electrodes were fixed at thenar and hypothenar sites of the non-dominant hand. For registration of respiratory activity, two PS-2 biofeedback respiration sensor belts (*KarmaMatters, Berkeley, CA, USA*) with a built-in length-dependent electrical resistance were used. They were fixed at the upper thorax and the abdomen. ECG was measured with *Hellige* electrodes (diameter 1.3 cm) according to Einthoven II. Finger pulse signal was transmitted by an infrared system in a cuff around the middle finger of the non-dominant hand.

### Behavioral measures

Subjects responded verbally as well as by pressing a key. Key presses indicating “yes” or “no” answers were time-logged, synchronized with the physiological measures, and stored on the stimulus-presenting computer. Importantly, answers were delayed by 4 s in this study; after this delay, most stimulus processing and answer preparation can be assumed to be completed; in addition, it is rather easy to perform strategic manipulations by voluntarily controlling reaction speed after the delay. Therefore, behavioral data were not analyzed. CIT questions with at least one item answered incorrectly were discarded from analysis, but no such case occurred.

### Questionnaire

As the last part of the experiment, participants filled in the *PPI-R*. It comprises 154 items to assess the individual degree of psychopathic traits; the sum scale and (for exploratory purposes) the nine subscales were calculated from the raw data and then, in order to account for gender differences, transformed into *T* values (Alpers and Eisenbarth, 2008). To investigate the relationship between the psychopathy measure and physiological responsiveness in the CIT, correlation coefficients were calculated for the individual *T* values on each subscale and the individual standardized *probe*-minus-*irrelevant* response differences for each physiological data channel.

### Data processing

Skin conductance data from two subjects (one from the *text* group, one from the *social* group) had to be discarded from analysis because of electrodermal non-responding. Skin conductance reactions were assessed by a computerized method (see Ambach et al., 2008; Ambach et al., 2010) based on the decomposition of overlapping reactions as proposed by Lim et al. (1997). This method was chosen because, two subsequent physiological reactions occurred with a short delay (due to the delay of 4 s between question and prompt to answer). With short interstimulus intervals, conventional trough-to-peak evaluation is inadequate (Lim et al., 1999), because the first of two reactions causes a diminishing bias in the estimation of the second one. The size of this bias is determined by the size of the first reaction and by the time interval between both reactions. Decomposition aims at overcoming this problem of overlapping EDA reactions.

After optimizing model coefficients for each subject, all trials were evaluated by decomposing EDA by use of the subject’s individual model coefficients. Then, magnitudes of all EDA responses that were elicited within a time window of 0.5 to 4.5 s after item presentation were additively combined to a “first response” (EDA_1). The sum of EDA responses, which began between 4.5 and 8.5 s after item presentation, i.e., between 0.5 and 4.5 s after the subjects were prompted to answer, was calculated as “second response” (EDA_2). For the regression analysis, a combined response measure (EDA_sum) was calculated by adding both components per trial. For each time window, the decomposed responses were transformed into their equivalent in μS according to the subject’s individual electrodermal response template.

Respiratory data were low-pass filtered (10 dB at 2.8 Hz); respiration line length (RLL) was automatically computed over a time interval of 15 s after trial onset. The RLL measure integrates information about frequency and depth of respiration. The method was derived from Timm (1982) and modified by Kircher and Raskin (2003). Respiratory data from one subject (from the *social* group) were discarded due to a technical failure. For analysis, raw data from both respiratory channels were averaged.

Electrocardiogram data obtained from three subjects (one from the *text* group, two from the *social* group) had to be excluded from analysis because of technical failure or arrhythmia. After notch filtering at 50 Hz, *R*-wave peaks were automatically detected and visually controlled. The *R*–*R* intervals were transformed into HR and real-time scaled (Velden and Wölk, 1987). The HR during the last second before trial onset served as pre-stimulus baseline. The phasic heart rate (pHR) was calculated by subtracting this baseline value from each second-per-second poststimulus value. For extracting the trial-wise information of the phasic HR, the mean change in HR within 15 s after trial onset, compared to the pre-stimulus baseline, was calculated (see Verschuere et al., 2007; Gamer et al., 2008).

Finger pulse waveform length (FPWL) data from five subjects (three from the *text* group, two from the *social* group) had to be discarded from analysis because of insufficient signal quality. The FPWL within the first 15 s after trial onset was calculated from the finger pulse waveform and then subjected to further analyses (Elaad and Ben-Shakhar, 2006). It comprises information about both HR and pulse amplitude.

In order to compare indicators of arousal between-groups, we additionally computed the individual averages of non-standardized SCL and HR at trial onsets. SCL and HR data were averaged over the last second before the onset of a CIT question (i.e., 3.5–4.5 s before item onset).

A within-subject standardization of measured values has been proposed by Lykken and Venables (1971). Here, according to Ben-Shakhar (1985), Gamer et al. (2006), and Gronau et al. (2005), the physiological measures are *z*-transformed for each subject and for each data channel. All *probe* and *irrelevant* trials (but not *neutral* trials and the first trials of each stimulus category) were used to calculate individual means and standard deviations. The *z*-transformed values were used in subsequent statistical analyses.

### Statistical analysis

Statistical analyses were performed with *SYSTAT*, Version 13 (*SYSTAT Software, Inc*., Monte Carlo).

For each measure, mean responses to *probe* vs. *irrelevant* items were compared using one-tailed *t*-tests (matched samples), separately for *text* and *social* group. An additional *t*-test was performed to test whether the *probe*-minus-*irrelevant* response differences were enhanced in the *social* as compared to the *text* group.

Significance level was set to 0.05; Cohen’s *d* was calculated as estimate of effect size (Cohen, 1988; Rosnow and Rosenthal, 1996).

Besides investigating the effects of the different questioning formats on each of the physiological measures, the capability of detecting concealed information in both groups was of interest from an applied perspective. For this purpose, the validity of each data channel and the validity of an optimized combination of the measures (EDA_sum, pHR, RLL, and FPWL) were analyzed using a binary logistic regression analysis. Because all participants in this study had deed-related knowledge, responses of a hypothetical group of “innocent” subjects were simulated according to Meijer et al., 2007; simulated trial-by-trial values were randomly drawn from a standard normal distribution.

Binary logistic regression analyses were performed with inclusion of each of the measures and with a fixed inclusion of all four measures (which in contrast to a stepwise inclusion prohibits that the included measures differ between-groups). A cross-validation was run using the hold-one-out method, separately for the *text* group and the *social* group: each subject’s classification as “guilty” or “innocent” was based on a combination of his or her standardized differential physiological responses with weights calculated from all other “guilty” and “innocent” subjects. The receiver-operating characteristic (ROC) allows to estimate the capability of differentiating guilty from innocent participants for all possible cut-off points and for different dependent measures and their combination. The area under the ROC curve varies between 0 and 1 with a chance level of 0.5 and serves as an overall index of detection accuracy (Bamber, 1975; Ben-Shakhar and Elaad, 2003; Gronau et al., 2005).

## Results

### Memory test

In the memory test, 99.2% of the *probe* items were identified correctly (99.0% in the *social* and 99.3% in the *text* group). Categories with false identification of the *probe* item were discarded from evaluation. (Note that restricting the analyses to categories with correct probe identification, as well as the exclusion of data from non-compliant or physiologically hyporesponsive participants, which are standard procedures within the experimental context, can lead to an inflation of effect sizes and detection rates when transferred to real-life CIT investigations.

### Overview of psychophysiological measures

Preceding data standardization and test statistics, descriptive statistics based on raw scores are presented. Table 1 summarizes means and standard errors of means of raw scores for each data channel separately for both groups.

**Table 1:** **Means and standard errors of means (SEM) of raw scores for each data channel**.

	*Text* group				*Social* group			
	*Probe* items		*Irrelevant* *items*		*Probe* items		*Irrelevant* *items*	
	Mean	SEM	Mean	SEM	Mean	SEM	Mean	SEM
EDA_1 (nS)	304	38	141	16	270	48	109	14
EDA_2 (nS)	320	41	233	33	283	34	186	22
pHR (1/min)	−1.41	0.45	0.41	0.24	−3.39	0.37	−0.90	0.30
RLL (arb. units)	1755	127	2019	151	1720	145	2005	163
FPWL (arb. units)	181	20	207	22	163	13	198	15

*Responses to *probe* and *irrelevant* items are listed separately for *text* and *social* group*.

**Figure 2:**
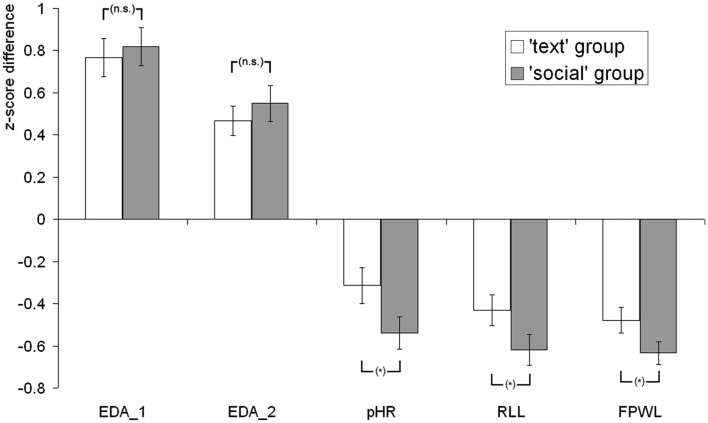
**Differential responses (*z*-scores) to *probe* vs. *irrelevant* items: for the *text* and the *social* group, standardized response differences are depicted for first electrodermal reaction (EDA_1), second electrodermal reaction (EDA_2), phasic heart rate (pHR), respiration line length (RLL), and finger pulse waveform length (FPWL)**. Error bars represent the SEM; *indicate the level of significance of the group difference (*text* vs. *social* group; “n.s.”: not significant; *p < 0.05).

Figure 2 illustrates the differential responses to *probe* vs. *irrelevant* items for both groups. Response differences (*z*-scores) between probe and irrelevant trials are depicted for each of the physiological measures.

### Skin conductance

**Figure 3:**
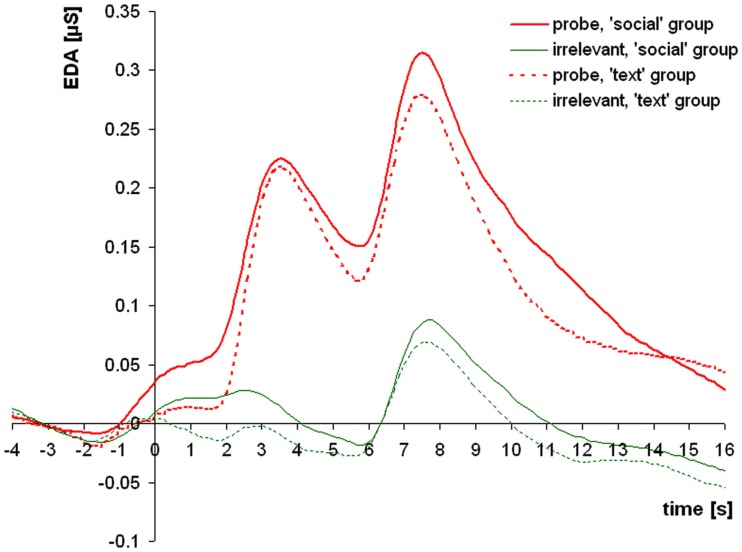
**Grand means of skin conductance responses to *probe* and *irrelevant* items for the *text* group and the *social* group**. After a small initial response to the appearance of the question (text or face and voice, respectively), two subsequent electrodermal responses of interest (EDA_1 and EDA_2) follow the item presentation and the prompt to answer.

Figure 3 shows the averaged intra-trial course of skin conductance depicting grand means for trials with *probe* and *irrelevant* items separately for both groups. The grand means show two strong EDA response components with an onset and peak asynchrony of 4 s, which is in accordance with the 4-s delay between item onset and prompt to answer. Response amplitudes to *probe* items exceeded those to *irrelevant* items by far in both groups, with no apparent difference between-groups. The additional EDA response, which was observed 3.5 s before the response to item onset, can be ascribed to the onset of the question text, or the face and voice respectively. An exploratory analysis of this component using *t*-tests (corresponding with the *t*-tests performed on all other measures) revealed no significant difference between item types and no group difference for the *probe*-minus-*irrelevant* differential response.

EDA_1 responses were greater to *probe* than to *irrelevant* items in the *text* group (*t*_32_ = 8.60; *p* < 0.001; *d* = 1.50) as well as in the *social* group (*t*_33_ = 9.01; *p* < 0.001; *d* = 1.55). The between-groups *t*-test for EDA_1 response differences did not reveal greater *probe*-minus-*irrelevant* response differences in the *social* as compared to the *text* group (*t*_65_ = −0.40; *p* > 0.1).

Analogously, EDA_2 responses were greater to *probe* than to *irrelevant* items in the *text* group (*t*_32_ = 6.77; *p* < 0.001; *d* = 1.18) as well as in the *social* group (*t*_33_ = 6.37; *p* < 0.001; *d* = 1.09). The between-groups *t*-test for EDA_2 response differences did not reveal greater *probe*-minus-*irrelevant* response differences in the *social* as compared to the *text* group (*t*_65_ = −0.74; *p* > 0.1).

An additional ANOVA for probe vs. irrelevant response differences, which included a factor “time” distinguishing between the first and the second electrodermal response component and a factor “group” distinguishing between text and social group, was performed. This analysis did not reveal an interaction between “time” and “group” (*p* > 0.1), indicating similar response patterns of the two electrodermal response components. For the logistic regression analysis, both components were then additively combined in a single measure: EDA_sum. EDA_sum responses were also greater to probe than to irrelevant items in the text group (*t*_32_ = 9.59; *p* < 0.001; *d* = 1.67) as well as in the social group (*t*_33_ = 9.48; *p* < 0.001; *d* = 1.63). Probe-minus-irrelevant response differences for EDA_sum were not greater in the social as compared to the text group (*t*_65_ = −0.41; *p* > 0.1).

### Respiration

Respiration line length values were smaller after *probe* than after *irrelevant* items in the *text* group (*t*_33_ = −5.93; *p* < 0.001; *d* = −1.02) as well as in the *social* group (*t*_33_ = −8.51; *p* < 0.001; *d* = −1.46). The between-groups *t*-test for RLL differences revealed greater *probe*-minus-*irrelevant* RLL differences in the *social* as compared to the *text* group (*t*_66_ = 1.82; *p* < 0.05; *d* = 0.44).

### Heart rate

Heart rate decelerations were more pronounced after *probe* than after *irrelevant* items in the *text* group (*t*_32_ = −3.64; *p* < 0.001; *d* = −0.63) as well as in the *social* group (*t*_32_ = −6.95; *p* < 0.001; *d* = −1.21). The between-groups *t*-test for pHR differences revealed greater *probe*-minus-*irrelevant* pHR differences in the *social* as compared to the *text* group (*t*_64_ = 1.94; *p* < 0.05; *d* = 0.48).

### Finger pulse

Finger pulse waveform length values were smaller after *probe* than after *irrelevant* items in the *text* group (*t*_31_ = 7.88; *p* < 0.001; *d* = −1.39) as well as in the *social* group (*t*_31_ = 11.82; *p* < 0.001; *d* = −2.09). The between-groups *t*-test for FPWL differences revealed greater *probe*-minus-*irrelevant* FPWL differences in the *social* as compared to the *text* group (*t*_62_ = 1.93; *p* < 0.05; *d* = 0.48).

### Tonic measures of arousal

When comparing indicators of arousal between-groups, SCL appeared higher in the *text* group (5.03 ± 2.41 μS) than in the *social* group (4.15 ± 1.66 μS). This was contrary to the expectation and would have reached statistical significance in case of an inverted *a* *priori* hypothesis (*t*_65_ = −1.730, *p* = 0.044). Inspection of the raw data indicated that this result was due to an initially enhanced EDA level in the text group that was preserved throughout the entire examination. HR appeared higher in the *social* group (76.02 ± 26.11 bpm) than in the *text* group (74.90 ± 21.53 bpm), but also this was not statistically significant (*t*_69_ = −0.196, *p* = 0.845).

### Receiver-operating characteristic

Binary logistic regression analyses were performed to classify the subjects (half “guilty” participants, and half hypothetical “innocents”) as “guilty” or “innocent”; hence, the *a priori* probability was set to 0.5. Separately for the *text* group and the *social* group, regression models were calculated with inclusion of each individual physiological measure as well as with fixed inclusion of EDA_sum, pHR, RLL, and FPWL. The classification performance was shrinkage-corrected using the hold-one-out method (which, in turn, resulted in different regression coefficients for each subject). The different rates of false-positive (classification of an “innocent” subject as “guilty”) and false-negative outcomes (classification of a “guilty” subject as “innocent”) obtained under variation of the cut-off point for decision were calculated separately for the *text* group and the *social* group.

Table 2 shows the areas under ROC and their confidence intervals for each of the single measures and the shrinkage-corrected areas under ROC for the optimal-weight combination of EDA_sum, pHR, RLL, and FPWL. (Note that for single measures the ROC values are equivalent to those obtained without a logistic regression analysis.)

**Table 2:** **Area under the receiver-operating characteristic (ROC) curves and 95% confidence intervals for a differentiation of guilty vs. hypothetical innocent subjects**.

Included parameters	Area under the ROC curve and 95% confidence intervals			
	*Text* group		*Social* group	
	Area	Confidence interval	Area	Confidence interval
***SINGLE MEASURES:***				
EDA_sum	0.885	0.784–0.965	0.901	0.821–0.965
pHR	0.742	0.618–0.862	0.813	0.698–0.906
RLL	0.748	0.627–0.860	0.832	0.724–0.922
FPWL	0.816	0.703–0.909	0.910	0.831–0.973
***OPTIMAL-WEIGHT COMBINATION:***				
EDA_sum + pHR + RLL + FPWL	0.922	0.845–0.977	0.971	0.919–1.000

*Shrinkage-corrected values are listed for an inclusion of each single physiological measure and for an optimal-weight combination of EDA_sum, pHR, RLL, and FPWL*.

**Figure 4:**
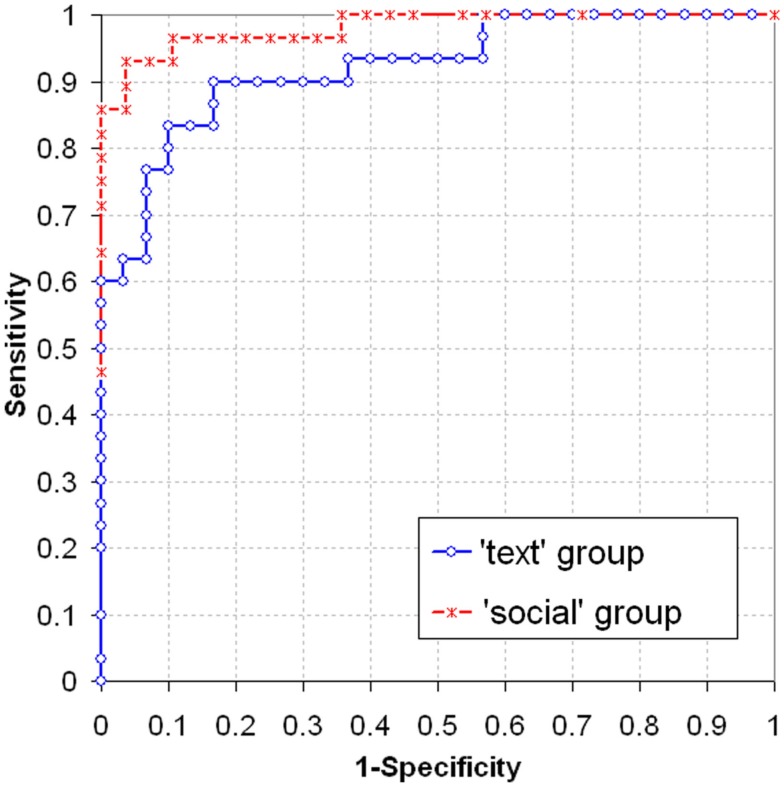
**ROC curves for *text* group and *social* group with the predictors EDA_sum, pHR, RLL, and FPWL**.

Figure 4 shows the ROC curves for the *text* group and the *social* group with the optimal-weight combination of the four physiological measures after shrinkage-correction.

While no difference between-groups in test validity is apparent with EDA, the single cardiovascular and respiration measures, and also the optimal-weight combination of measures, yielded apparently greater areas under ROC for the *social* group than for the *text* group. According to the large confidence intervals however, none of these between-groups differences turned out significant in a bootstrap analysis (*p* > 0.05 for FPWL; *p* > 0.1 for all other measures and for the optimal-weight combination).

### Psychopathic personality inventory – revised

From the *PPI-R*, the individual sum scores and (for exploratory purposes, not reported) the nine subscale scores were calculated. *PPI-R* data from the two participants precluded from physiological analysis were treated as missing data.

For female participants (*N* = 37), the *PPI-R* sum scores of 277.65 ± 21.49 were lower than in the reference sample (313.33 ± 25.45; Alpers and Eisenbarth, 2008; *p* < 0.001). For male participants (*N* = 32), the sum scores of 299.32 ± 27.25 were also lower than in the reference sample: 325.42 ± 24.92; *p* < 0.001).

Correlation coefficients between *T*-transformed *PPI-R* sum scores and *probe*-minus-*irrelevant* response differences for each physiological data channel were calculated for the *social* and the *text* group and across groups. Correlation coefficients for EDA_sum were 0.05 across groups, −0.01 for the social and 0.12 for the text group. The according values were −0.09, 0.00, and −0.18 for pHR, −0.03, −0.01, and −0.05 for FPWL, and −0.10, −0.01, and −0.20 for RLL, respectively. None of these correlations was statistically significant (all *p* > 0.1, uncorrected), indicating that differential responding in the CIT was not found to be moderated by *PPI-R* sum scores in either group or across groups. An additional bootstrap analysis was performed to assess confidence intervals for the correlation coefficients in either group; none of the group differences in correlation coefficients was significant (*p* > 0.1 each, uncorrected), which suggests that the enhanced differential responding found in the social group for pHR, RLL, and FPWL was not moderated by *PPI-R* sum scores.

## Discussion

Social influences on physiological responding in the CIT are insufficiently explored. The present study compared two computer-based CIT conditions with respect to differential physiological responses: one condition used a text-based interrogation; the other included face and voice as social stimuli into the interrogation. A psychopathy questionnaire was administered in order to investigate personality influences on physiological responding and to explore a possible interaction of psychopathy with the impact of the social stimuli.

### Overall CIT effects

For each physiological measure, in either of the two interrogation conditions as well as across conditions, significant response differences with large effect sizes were found between *probe* and *irrelevant* items.

Finger pulse waveform length yielded the greatest overall effect size, which is somewhat uncommon among most CIT studies. Elaad and Ben-Shakhar (2006), however, reported “that detection accuracy with the FPWL was at least as good as the accuracy obtained with (…) respiration changes and skin conductance responses.” Similarly, Vandenbosch et al. (2009) found CIT accuracy with FPWL as high as with EDA and better than with RLL, pHR, and finger pulse amplitude. Given that an adequate scoring of FPWL depends on sufficient signal quality, it might well be that influences such as the surrounding temperature before the experiment or the delay between temperature customization and CIT initiation, which are commonly not reported, differently influence finger pulse amplitude (and thereby signal quality and CIT accuracy) in different studies.

The time courses of skin conductance showed two temporally distinct response components with a delay of 4 s. Both EDA components, one after stimulus presentation, the other after the prompt to answer, showed large response differences between *probe* and *irrelevant* trials, with the first component yielding the greater effect size, which is in line with earlier studies (e.g., Ambach et al., 2008).

From a “detection” perspective, each of the measures in either group was capable of significantly differentiating “guilty” from hypothetical “innocent” subjects; ROC area values are in line with other studies. With an optimized linear combination of measures, the ROC area was 0.946 across groups, which reflects an adequate overall CIT accuracy.

### Text condition vs. face and voice condition

In a between-subjects manipulation, two CIT conditions differed in the way the CIT questions were presented (*text* group: text on the screen; *social* group: voice via speakers plus face on the screen); depiction of CIT items was identical.

Differential responding in pHR, RLL, and FPWL, indicating cardiac, pulmonary, and vascular functioning, differed significantly between conditions, which met the *a priori* expectation: differential responding to *probe* vs. *irrelevant* items was enhanced in the condition with social stimuli, as compared to the text presentation. Contrary to our expectation, differential electrodermal responses did not mirror this finding; electrodermal response differences neither differed significantly between conditions for the first nor the second component (nor for the two components combined).

The classification of “guilty” and (hypothetical) “innocent” subjects by means of an optimized linear combination of standardized measures yielded ROC area values of 0.922 in the *text* group and 0.971 in the *social* group. Although the difference in curves between conditions appears prominent visually, and although the size of the underlying group effect differed significantly between-groups for three of the measures, this group difference in ROC curves was not statistically significant. The inclusion of data from “innocent” participants (be they real or hypothetical) entailed additional error variance which obscured the significant group difference observed in the dependent measures.

### Possible explanations and confounds

Given the different impact the two-part experimental manipulation (combining face and voice in the *social* condition) exerted on the physiological measures, it can be conjectured once more (see e.g., Ambach et al., 2008, 2011b) that the responses of the individual physiological channels are not reflecting a singular psychophysiological process ongoing in the CIT (such as a unitary orienting response; see Barry, 1996, 2006). The observation that all physiological measures, except the electrodermal, were affected by the experimental manipulation suggests that processes other than orienting seem to depend on the type of CIT presentation. Earlier CIT studies, which were based on the electrodermal measure (e.g., Gustafson and Orne, 1963; Elaad and Ben-Shakhar, 1989; Furedy and Ben-Shakhar, 1991; Ben-Shakhar and Elaad, 2003), document that intentional and motivational manipulations influenced differential responding also for EDA. One might conclude that this renders intention and motivation less likely the moderators of the manipulation observed in the present study. A difference between conditions in physiological measures of arousal, which might have contributed to an explanation, remained also unproven. It is further conceivable that the different presentation types directed a participant’s attention in a different manner; a voice might more forcefully direct attention to the item presented thereafter, whereas a displayed text might allow to divert attention more easily. The longer duration of voice presentation, as compared to capturing a written text, might contribute to this. An additional, more speculative explanation, refers to experimental attempts to disentangle orienting from deceptive components in a CIT (Ambach et al., 2008; Matsuda et al., 2012). The instruction to deceptively deny specific knowledge had greater effects on cardiovascular and/or respiratory measures than on EDA. One might thus speculate that face and voice, in contrast to a visual text presentation, affect the same CIT subprocess reflected in pHR, RLL, and FPWL, namely a subprocess closely related to deceptive action. Finally, the failure of EDA to replicate the findings of the other measures might be explained by a ceiling effect; in case the electrodermal system is maximally differentially activated already with textual question presentation, then social stimuli cannot be expected to enhance differential responding. If such a ceiling effect is assumed, then a “face and voice” presentation might be particularly advantageous over a “text only” presentation when measures other than EDA are used, or when EDA, due to suboptimal test conditions, does not reach optimal detection levels (e.g., in cases of only partial crime-related knowledge of the suspects, or with the use of countermeasures).

As Bradley (2009) suggested, the orienting response can fruitfully be regarded as embedded in motivational and attentional systems active and fluctuating within an individual. Instead of debating whether the “social” manipulation in this study had an impact on orienting, motivation, attention, or emotion, it might be more groundbreaking to regard the presence or absence of social stimuli as modifying the subject’s environment in the sense that it alters the intentional, motivational, attentional, and emotional background the orienting response takes place in.

Whether it was the virtual investigator’s face or his voice presented via speakers, or the combination of both, that determined the enhancement of differential responding in the affected measures, cannot be decided from this study: following the primary aim to maximize the experimental manipulation, face and voice were planned to occur in fixed combination.

A confound of the text vs. voice question presentation, meant to contrast absence vs. presence of voice as a social stimulus, with the visual vs. auditory presentation modality is obvious. This confound is inherent whenever visually presented text is compared with spoken word: speech is essentially human; therefore, spoken text is always a social stimulus (even in case of an alienated voice). Thus, if an auditory presentation (even without face) enhanced differential responding more than a visual text presentation, the question whether this may be called a “social” effect seems subordinate to the question, by which pathways spoken text is superior to written text in the CIT.

### Personality aspects: Psychopathy and the CIT

In line with earlier studies, *PPI-R* scores were greater in male than in female participants. However, the overall scores (for both genders) were smaller than the normative values for the German questionnaire version (Alpers and Eisenbarth, 2008). On the other hand, Uzieblo et al. (2010) provided standard sum scores obtained from a large population sample (males: 283.00 ± 34.30, *N* = 419; females: 266.87 ± 32.12, *N* = 256). Taking these values into account (although obtained with a different translation of the *PPI-R*), the mean values obtained in the present study do not point toward a biased sample.

We did not find a correlation between the psychopathy sum score and differential physiological responding in any of the four measures; the results of explorative analyses with subscales are not reported due to their complexity and fruitlessness. Likewise, no interaction effect was found that would have pointed toward a different impact of the social stimuli in individuals with high or low psychopathy scores.

### Suggestions for laboratory studies

In follow-up research, the two parts of the experimental manipulation should be disentangled. Voice presentation of CIT questions should be compared with a visual text presentation, separately from investigating the influence of additional social stimuli such as a face; a follow-up study should enrich the present design by including a condition with auditory question presentation but without a depicted face.

While it should be easy to separate the influences of face from the influence of voice, it might not be that easy to separate modality effects (i.e., auditory vs. visual question presentation) due to the social character of the presentation (social: human voice; non-social: written text); this is due to the social nature of speech, *per se*.

Beyond the social stimuli used in the present study, the importance of particular elements of social presence and interaction should be highlighted more in CIT research. Beside the impact of isolated social stimuli (e.g., a depicted pair of eyes, or the sounds of a human voice), the importance of an investigator’s appearance, demeanor, and social acting deserves more attention. This will resume a line of research that lay idle for a couple of decades (see e.g., Waid and Orne, 1981), due to the desire to standardize experiments as far as possible. Balancing standardization of experimental conditions and the investigation of social influences on psychophysiology in the CIT, which can be standardized only to a limited extent, should be aimed at. An additional suggestion arises from the design of the present study. Instead of the between-subjects design used here, conditions differing with respect to “social content” could be compared within-subject, given that this can be implemented meaningfully.

### Suggestions for applied research

Besides inferable clear guidelines for future laboratory studies, the observed results have implications for research on the field application of the CIT. Different CIT interrogation types, different CIT settings, as well as different experimenter roles (e.g., harshness, social support, expression) and other specific characteristics (e.g., gender, ethnicity) might have a different social impact on a person investigated in the CIT (see Iacono, 2000). Studying these influences, comparing different practical settings, and optimizing conditions with respect to test accuracy, should become a focus of CIT research again. This might resolve left open and since the early eighties of the last century neglected questions. In the same vein, advantages and shortcomings of computer-based CIT interrogations might be focused on in more detail.

As a methodological suggestion, application-oriented CIT research should include the investigation of “innocent” (unknowledgeable) participants. The binary logistic regression and ROC analyses applied in this study were done to compare detection accuracy between conditions, although this was not the primary aim of the study. Synthesizing a group of hypothetical “innocent” participants for this purpose is of limited value: due to the additional random procedures involved in supplementing data for “innocents,” statistical significance of ROC comparisons clearly remains below that of the *probe* vs. *irrelevant* effects calculated from actually collected data. Depending on the details of generating simulated data, distortions might also be entailed, e.g., by disregarding distributions or mutual correlations of physiological data (leading to overestimated ROC areas for combined measures). In future CIT studies which go beyond the “effect size” perspective and focus detection accuracy instead, unknowledgeable participants should be included.

## Conclusion

A uniform male face presented with every question and item in a CIT, together with an auditory instead of a visual presentation of the question text, enhanced differential responding in a CIT in several physiological measures but not EDA. Taking possible confounds into account, the present study provides evidence that beyond the mere presentation of items about which knowledge has to be deceptively denied, influences of social stimuli seem to play an important role in the CIT. The social situation, in which the CIT takes place, should receive more attention in future research and application. Besides focusing on practical matters, further studies should disentangle the influence of emotional content of the social situation (e.g., friendly, controlling, antagonistic), specific elements of social interaction (e.g., personal questioning, evaluative watching, mere presence), and presentation modality.

## Conflict of Interest Statement

The authors declare that the research was conducted in the absence of any commercial or financial relationships that could be construed as a potential conflict of interest.
